# The Role of Cancer-Associated Fibroblasts in Hepatocellular Carcinoma and the Value of Traditional Chinese Medicine Treatment

**DOI:** 10.3389/fonc.2021.763519

**Published:** 2021-11-18

**Authors:** Wentao Jia, Shufang Liang, Binbin Cheng, Changquan Ling

**Affiliations:** ^1^ School of Traditional Chinese Medicine, Naval Medical University, Shanghai, China; ^2^ Department of Traditional Chinese Medicine, Changhai Hospital, Navy Medical University, Shanghai, China

**Keywords:** cancer-associated fibroblasts (CAFs), traditional Chinese medicine, hepatocellular carcinoma (HCC), stromal cell, tumor recurrence

## Abstract

Invasion and metastasis are the main reasons for the high mortality of liver cancer, which involve the interaction of tumor stromal cells and malignant cells. Cancer-associated fibroblasts (CAFs) are one of the major constituents of tumor stromal cells affecting tumor growth, invasion, and metastasis. The heterogeneous properties and sources of CAFs make both tumor-supporting and tumor-suppression effects possible. The mechanisms for CAFs in supporting hepatocellular carcinoma (HCC) progression can be categorized into upregulated aggressiveness and stemness, transformed metabolism toward glycolysis and glutamine reductive carboxylation, polarized tumor immunity toward immune escape of HCC cells, and increased angiogenesis. The tumor-suppressive effect of fibroblasts highlights the functional heterogenicity of CAF populations and provides new insights into tumor–stromal interplay mechanisms. In this review, we introduced several key inflammatory signaling pathways in the transformation of CAFs from normal stromal cells and the heterogeneous biofunctions of activated CAFs. In view of the pleiotropic regulation properties of traditional Chinese medicine (TCM) and heterogeneous effects of CAFs, we also introduced the application and values of TCM in the treatment of HCC through targeting CAFs.

## Introduction

Hepatocellular carcinoma (HCC), one of the deadliest malignant diseases, claims more than 2,814,000 lives a year in China (1). To date, the 5-year survival rate of HCC patients after hepatectomy is still below 60% (2, 3). The recurrence and metastasis of HCC after radical resection account for more than 90% cases of early postoperative death (4). The dismal curative ratio of HCC has yet to be significantly improved despite several emerging therapies, which poses a great challenge to clinicians and researchers.

Currently, the therapeutic targets have been transformed from the tumor itself to the stromal cells in the tumor microenvironment (TME), which includes fibroblasts, endothelial cells, mesenchymal cells, and immune cells. HCC progression does not completely depend on tumor autonomous signaling (5). The cross-talks between stromal cells and cancer cells, as well as the communication among different kinds of stromal cells, are of great significance in constructing tumorigenic environments (6), which account for the dismal prognosis of HCC. Such interactions have gained much popularity in the treatment of HCC. Fibroblasts are one of the major constituents of tumor stromal cells, which originate from a variety of cell types in the presence of complex signaling. Several studies have identified components including tissue residual fibroblasts, endothelial cells undergoing endothelial-to-mesenchymal transition (EndMT), pericytes, vascular smooth muscle cells, cancer cells that undergo the epithelial–mesenchymal transformation (EMT), and bone marrow-derived cells (BMDCs) as the origins of cancer-associated fibroblasts (CAFs) (6, 7).

The multiple origins and diverse functions of CAFs make the heterogeneity of cell populations and distinct effects on tumor growth. With insufficient biomarker distinguishing all subtypes of fibroblasts, the identification of CAFs with different functions requires further investigation (8). Overall, the main types of CAFs exert a tumor-supporting effect. Several clinical studies have confirmed the tumor-supporting effect of CAFs and demonstrated that abundant CAFs in HCC tissue always predict poor prognosis after radical operation (9–11). Oddly, in some studies, the upregulation of CAF level in HCC tissue does not necessarily indicate poor prognosis, which means that some subtypes of CAFs as well as their cytokines and metabolic products show little effect on the progression of HCC and may even suppress the malignance of the tumor (12–17). Also, the neoplasm and metastasis lesions are commonly encased by fibrotic tissue and collagen, suggesting that tumor progression can be restrained by desmoplasia and some CAF populations (13, 18). In a cohort study, the high expression of fibroblast activation protein (FAP) is not associated with the disease-free survival time (DFS) of HCC patients after hepatic resection (16). This contradictory phenomenon merits long-term clinical studies with larger samples and more high-quality fundamental studies.

In this review, we introduced several key inflammatory signaling pathways in the transformation of CAFs from normal stromal cells, the metabolism reprogramming of activated CAFs, and the heterogeneous biofunctions of activated CAFs on HCC progression, metastasis, and recurrence. Finally, we also introduced the application and values of traditional Chinese medicine (TCM) in the treatment of HCC through targeting CAFs.

## The Activation of CAFs in the Premalignant Microenvironment of HCC

**Figure 1:**
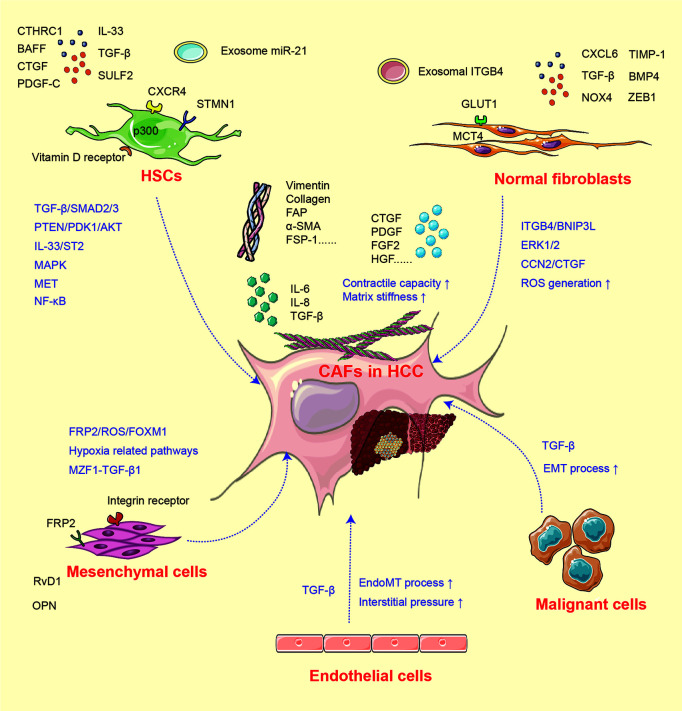
The activation of CAF from heterogeneous cells.

In fibrosis and cirrhosis, the premalignant microenvironments are considered especially significant under the situation of tissue injury, hypoxia, and chronic inflammation. As tumors are regarded as “wounds that never heal”, the activation of fibroblasts is a critical process in tumor progression (19). Generally, fibroblasts in the liver environment are mainly distributed around the fibrous septum, fibrous capsule, and hepatic blood sinusoids and activated in the existence of tumor-associated signals (8, 19). Quiescent hepatic stellate cells (HSCs) and other normal fibroblasts suppress malignant cell growth *via* contact inhibition (18). While under liver injury, the inflammatory factors including sulfatase (SULF)-2, transforming growth factor (TGF)-β, CXCL6, and CXCR4 educate normal fibroblasts and other relevant cells toward CAFs (20–22) and increase the expression of CAF markers such as α-smooth muscle actin (SMA), FAP, vimentin, collagen, and fibroblast specific protein (FSP)-1 (23). The mechanisms for CAF activation are summarized in **Figure 1**.

CAFs in HCC have heterogeneous cellular origins (7). As mentioned above, multiple populations undergoing mesenchymal transition and equipped with fibroblastic property can develop into CAFs under inflammatory signal. Quiescent HSCs are the major source of CAFs, and the activation of HSCs is established as a pivotal step of liver fibrosis and tumor initiation. Under normal situation, vitamin D receptor in HSCs is stimulated by p62/SQSTM1 (24), while under pathological state, this process is abolished due to the loss of p62, which induces HSCs into CAFs and further supports HCC initiation (24). In addition, numerous studies have investigated the HCC–HSC feedback loop. Co-culture with HCC cells enhances the expression of CAF biomarkers α-SMA, vimentin, FAP, and FSP-1 in HSCs (23). The conditioned medium collected from activated HSCs can in turn stimulate tumor cell proliferation, stemness, and invasion (18). TGF-β is considered as the most potent signal involved in HSC activation (21, 23, 25). The activation of the TGF-β/SMAD pathway induces type I and III collagen expression in human HSC line LX2. It has been confirmed that SULF2 expressed by HCC cells can induce HSCs into CAFs through the TGF-β/SMAD3 pathway, which may further inhibit apoptosis and promote EMT of HCC cells *via* the SDF-1/CXCR4-related signaling pathway (20). p300 acetyltransferase promotes TGF-β-stimulated HSC activation by both cytoplasm-to-nucleus shuttle for SMAD2/3-tafazzin (TAZ) and histone acetylation mechanisms (26). Recently, it was reported that HCC-derived SDF-1 upregulates the expression of TGF-β and activates LX2 into CAFs (27). Meanwhile, TGF-β can also activate mitogen-activated protein kinase (MAPK) signals, further endowing the tumor-supporting property of HSCs (6). Apart from TGF-β, factors including platelet-derived growth factor (PDGF)-C, microRNA (miRNA)-21, interleukin (IL)-33, CTHRC1, and bone morphogenetic protein (BMP)-4 can also activate HSCs and facilitate liver fibrosis (28–32).

In addition to HSCs, components including mesenchymal stem cells (MSCs), hepatic sinus endothelial cells (HSECs), and even tumor cells can be developed into CAFs. In a hypoxic microenvironment, MSCs are transformed toward CAFs (31, 33, 34), then enhance the expression of cyclooxygenase (COX)-2 and prostaglandin E2 (PGE2), and subsequently facilitate HCC progression (31). Another study found that after co-cultured with SK-Hep1 cells, the expression of CAF biomarkers tenascin-C and SDF-1 is significantly upregulated in human MSCs (34). The MSC-derived CAFs then facilitate the EMT process of SK-Hep1 cells through integrin-related signaling pathways. The high expression of osteopontin (OPN) in a cancer microenvironment can mediate the MSC–CAF transition *via* binding with the integrin receptor and stimulation of the downstream MZF1–TGF-β1 pathway (33). Additionally, cancer-derived exosomes can trigger the process of EndMT in HSECs. The transformed HSECs display CAF features and express increased CAF biomarkers (35). HCC cells located around the blood sinusoid and undergoing EMT commonly express the CAF biomarkers α-SMA and FAP, indicating that both TGF-β and hypoxia-inducible factor (HIF)-1α enriched under hypoxic condition can induce CAF features in HCC cells (22, 36).

Interestingly, the activation of CAFs is reversible. It has been proven that chronic hypoxia may deactivate CAFs, reduce contractile force and stiffness in the surrounding extracellular matrix, and thereby alleviate cell invasion (37). The reversible characteristics of CAFs may provide therapeutic targets and new mechanisms for HCC progression. The molecular factors and signaling pathways for CAF activation are listed in **Table 1**.

**Table 1:** The factors and pathways for CAF activation in the tumor/premalignant microenvironment.

Cell origins	Factors	Signaling pathways and/or mechanism	Phenotypes and/or biomarkers	Reference
HSCs	SULF2	TGF-β1/SMAD3	α-SMA, FAP, and POSTN ↑	(20)
	TGF-β	TGF-β1/SMAD3	α-SMA, FAP, and collagen ↑	(21, 38, 39)
	CXCR4	SDF-1/CXCR4/TGF-β	α-SMA, vimentin, FSP1, and FAP ↑	(40)
	p300	TGF-β1/SMAD2/3-TAZ and histone acetylation	CTGF, tenascin-C, POSTN, PDGF-C, and FGF2 ↑	(26)
	IL-33	IL-33/ST2 and MAPK	IL-6, TGF-β, α-SMA, and collagen ↑	(41)
	PDGF-C		Desmin and αSMA ↑	(29)
	Exosomal miRNA-21	PTEN/PDK1/AKT	α-SMA, VEGF, MMP2, MMP9, bFGF, and TGF-β ↑	(28)
	CTHRC1	TGF-β	α-SMA and migratory and contractile capacities ↑	(32)
	BAFF	NF-κB	α-SMA and FAP ↑	(42)
	STMN1	MET	α-SMA, FSP, and HGF ↑	(43)
	CTGF		α-SMA and IL-6 ↑	(44)
	p62/SQSTM1	Vitamin D receptor binding	α-SMA, collagen, TGF-β, and PDGF ↓	(24)
Stromal fibroblasts	MCT4		GLUT1 and lactate production ↑	(45)
	Exosomal ITGB4	ITGB4/BNIP3L	Lactate production ↑	(46)
	CXCL6 and TGF-β	ERK1/2	CLCF-1 ↑	(21)
	NOX4	Intracellular ROS generation and TGF-β	α-SMA and collagen 1 ↑	(37, 47)
	TIMP-1	MMP inhibition	α-SMA, FAP, and vimentin ↑	(48)
	BMP4		ACTA2, COL1A1, IL-6, IL-8, and CCL2 ↑	(49)
	ZEB1, CTGF	CCN2/CTGF	α-SMA and ZEB1 ↑	(44, 50)
Mesenchymal cells		Hypoxia	COX2 and PGE2 ↑	(31)
	RvD1	FPR2/ROS/FOXM1	COMP and FOXM1 ↓	(51)
	OPN	OPN–integrin interaction and MZF1-TGF-β1	SMA, tenascin-C, vimentin, FSP-1, and MMPs↑	(33, 52)
Endothelial cells	Interstitial pressure and exosomal TGF-β	Endothelial to mesenchymal transition	α-SMA, FSP-1, and angiogenesis ↑	(35)
HCC cells	TGF-β	EMT	α-SMA and FAP ↑	(22, 36)

HSC, hepatic stellate cell; SULF2, sulfatase 2; TGF-β: transforming growth factor-β; SMAD, *Drosophila* mothers against decapentaplegic protein; CXCL, C-X-C chemokine ligand; α-SMA, α-smooth muscle actin; FAP, fibroblast activation protein; POSTN, periostin; SDF-1, stromal cell-derived factor-1; FSP, ferroptosis suppressor protein; CTGF, connective tissue growth factor; FGF, fibroblast growth factor; IL, interleukin; MAPK, mitogen-activated protein kinase; PTEN, phosphatase and tensin homolog deleted on chromosome ten; PDK1, 3-phosphoinositide-dependent kinase-1; AKT, protein kinase B; VEGF, vascular endothelial growth factor; MMP, matrix metalloproteinase; NF-κB, nuclear factor-κB; GLUT, glucose transport-1; ITGB4, integrin-β4; ROS, reactive oxygen species; TIMP-1, tissue inhibitor of metalloproteinases; OPN, osteopontin; EMT, epithelial–mesenchymal transition. ↑：upregulation, ↓：downregulation.

## The Mechanisms for CAFs in Supporting HCC Progression

Several studies have demonstrated the prognostic value of CAFs in predicting recurrence and metastasis of HCC after radical therapies. The high levels of CAFs as well as released proteins are commonly correlated with worse postoperative prognostic indices including early recurrence and metastasis; shortened DFS, OS, and PFS; and low survival rate (9–11), except for rare cases (12–17). In living donor liver transplantation, recipients with high levels of α-SMA+ CAFs in HCC display poorer prognosis (11). The high levels of CAF-related fibroblast growth factor (FGF)-2, endostatin, and VEGF may predict HCC recurrence (53). In HCC patients who underwent curative resection, factors including FGF3, FGFR2, Lysyl-oxidase-like (LOXL)-2, klotho-β, and FGF19 indicate a higher risk of tumor recurrence and worse survival rate. In patients who underwent HCC ablation, the level of serum FGF19 can serve as a potential prognostic biomarker (54–57).

**Figure 2:**
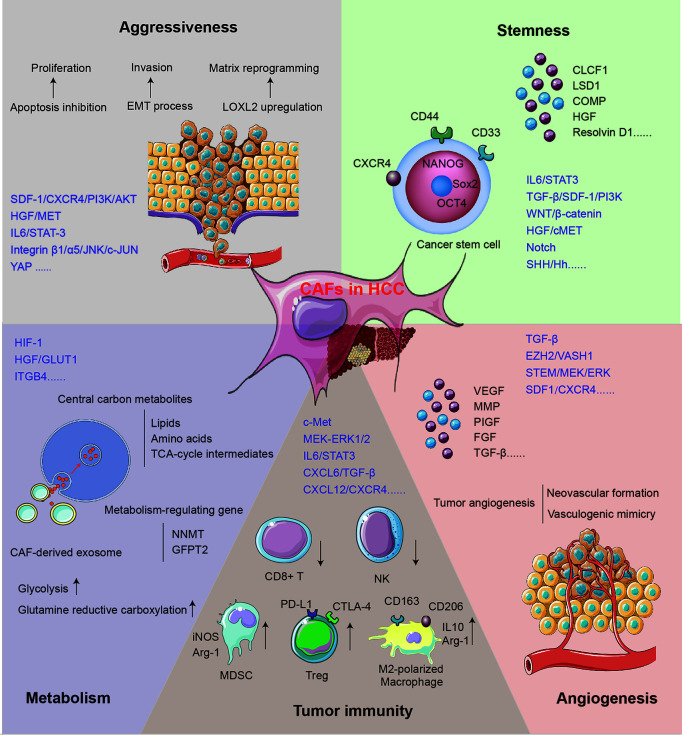
The mechanisms for CAFs in supporting HCC progression.

The mechanisms for CAFs supporting tumor growth can be acknowledged as the direct influence on HCC cells and the reformation of the microenvironment. The role of CAFs in supporting HCC progression is concluded in **Figure 2**.

### CAFs Promote the Aggressiveness of HCC Cells

The aggressiveness of HCC manifests in the upregulated proliferation and motility of cancer cells. SDF-1, one of the cytokines secreted by CAFs, may inhibit the apoptosis of HCC cells *via* the SDF-1/CXCR4/PI3K/AKT axis (48) and induce EMT through the SDF-1/CXCR4/OIP5-AS1/miR-153-3p/SNAI1 axis (20). Both classical and non-classical EMT processes play key roles in regulating tumor invasion and migration, which can lead to tumor recurrence and metastasis (58). Once treated with the conditioned medium of activated MRC5, the EMT phenotype was enhanced in human HCC cell lines Bel-7402 and LM3. Classical EMT markers including Snail, Twist, and integrin were regulated, while non-classical markers like E-cad/β-catenin complex, α/γ-catenin, and p120 catenin were redistributed in cells (58). Another study found that stathmin (STMN)-1 is highly expressed in HCC tissue, serving as a negative prognostic biomarker and mediating HCC recurrence and chemoresistance (43). STMN1 can facilitate the communication between CAFs and HCC *via* the HGF/MET pathway (43, 59). The IL-6/STAT3 signaling pathway is one of the most important mechanisms mediating HCC aggressiveness. Upregulated connective tissue growth factor (CTGF) in fibrosis environment can stimulate the IL-6 release from HSCs and accelerate HCC progression *via* the EMT process (44). Chemoresistance serves as another significant mechanism mediating HCC progression and metastasis after radical surgery. A recent study found that co-culture with CAFs maintains sorafenib resistance in HCC cells through B-cell-activating factor (BAFF)/NF-κB-dependent pathway (42); otherwise, overexpressed immunosuppressive cell differentiation (CD)-73 in cancer cells induced by CAFs can facilitate the resistance of sorafenib and cisplatin in HCC patients (60, 61).

CAFs can remodel ECM to gain cancer-supporting properties, and the function includes matrix degradation, deposition, and stiffening. Recently, tumor matrix stiffness is becoming an emerging target for tumor-aggressiveness study. Under high matrix stiffness condition, HCC releases a high level of LOXL2 through the integrin β1/α5/JNK/c-JUN signaling pathway. LOXL2 secreted by HCC can facilitate the formation of lung premetastatic niche *via* CD11b+ CD45+ BMDC recruitment, as well as fibronectin and matrix metalloproteinase (MMP)-9 upregulation (54, 62). A clinical study has confirmed the relationship between a high level of LOXL2 and worse prognosis in patients who underwent HCC radical surgery (54). Furthermore, overexpressed CXCR4 induced by high matrix stiffness can decrease the level of UBTD1 and promote proteasome-dependent degradation of YAP, contributing to HCC aggressiveness *via* the YAP signaling pathway (31). In addition, high matrix stiffness can promote the expression of the EMT transcription factor Snail through following three signaling pathways: integrin-mediated S100A11 membrane translocation, eIF4E phosphorylation, and TGF-β1 autocrine. The enhanced EMT process contributes to increased malignancy and progression of HCC (63).

### CAFs Induce Stemness of HCC Cells

The residual cancer cells at the margins of the resection area are generally recognized as the main cause of HCC recurrence and metastasis. The liver cancer stem cells (LCSCs) are characterized by self-renewal and multilineage differentiation and play a key role in the chemoresistance, metastasis, and recurrence of HCC (64, 65). The stemness is stimulated by signals from TME including GSK-3β/β-catenin, Notch, Wnt, and IL-6/STAT3 (64, 66, 67). Recently, accumulating studies have evaluated the role of CAFs in HCC stemness induction. In the CAF–LCSC feedback loop, LCSCs can bolster the proliferation and transformation of CAFs. On the other hand, CAFs can in turn induce and maintain the stemness of HCC through the IL-6/STAT3, TGF-β/SDF-1/PI3K, Wnt/β-catenin, HGF/cMET, and SHH/Hh pathways (23, 68).

HGF, a pivotal factor in the regulation of liver fibrosis and hepatocyte growth, is extensively studied in CAF–HSC communication. Studies showed that the HGF expressed by CAFs can induce stemness through the MET-STEM and ERK1/2–FRA1–HEY1 signaling pathways (10, 69). Among other CAF-secreted factors, CLCF-1 increases the secretion of CXCL6 and TGF-β by HCC cells and promotes HCC stemness through autocrine effects (21). The nuclear Notch intracellular domain in HCC tissues, which symbolizes the activation of tumor stemness, is correlated with poor prognosis (70). The activated Notch signaling pathway can be attributed to the stimulation of high CAF-derived IL-6 and downstream STAT3 phosphorylation (70). Furthermore, Notch can also be stimulated by CAF-derived chromatin modification factor LSD1 (71). Another study showed that COMP secreted by CAFs can induce stemness and EMT in HCC cells, which can be impeded by an endogenous anti-inflammatory lipid mediator Resolvin D1 treatment *via* FPR2/ROS/FOXM1 (51). Changed matrix stiffness caused by CAFs can also promote the release of HGF *via* signals including ERK, PKB/Akt, and STAT3, which can further induce the stemness of HCC (72).

Not only CAFs but also peri-tumor fibroblasts can maintain the stemness of HCC cells. Studies showed that peri-tumor fibroblasts express more IL-6 than CAFs, which can promote stemness in an IL-6–STAT3-dependent manner (73). Interestingly, peri-tumor fibroblasts can also exert tumorigenesis effect and transform normal liver cells into malignant tumor cells (73). In addition to stemness induction effect, CAFs and peri-tumor fibroblasts can recruit cancer stem cells by releasing cytokines, including IL-6, CCL2, CXCL1, and CXCL8 (74). Peri-tumor fibroblasts can also facilitate intrahepatic metastasis *via* SCGF-β- and HGF-mediated signaling pathways (74).

### CAFs Regulate the Metabolism in Tumor Cells

The aberrant metabolism of glucose, lipid, protein, and oxygen is one of the hallmarks of HCC, which constitutes a selective advantage for tumor growth, invasion, and metastasis. It is well recognized that CAFs can reprogram energy metabolism in HCC and promote the malignant phenotype of HCC including EMT, LCSC-like property, and drug resistance (75). In HCC, metabolic coupling, which happens between catabolic CAFs and anabolic cancer cells, provides mitochondrial fuels for oxidative phosphorylation and drives higher disease stage and shorter DFS *via* glycolysis (76). Stromal–epithelial metabolic coupling can be initiated through downregulation of HGF, which was demonstrated by decreased glucose transporter (GLUT)-1 level and reduced glucose uptake in cancer cells (77). Furthermore, the regulation of GLUT level may influence the production of lactate and remodel the metabolic pattern (45, 77). This metabolic reprograming can be mediated by the HIF-1-dependent signaling pathway, leading to the energic metabolism dominated by lactic acid rather than glucose (76).

The exosome derived from CAFs provides central carbon metabolites including amino acids, lipids, and tricarboxylic acid (TCA)-cycle intermediates for HCC cells in a micropinocytosis manner, which can inhibit mitochondrial oxidative phosphorylation and promote glycolysis and glutamine-dependent reductive carboxylation in tumor cells. This mechanism makes it possible for tumor cells to proliferate and migrate in an energy-deprived environment (75). Furthermore, a study has confirmed that integrin subunit β (ITGB)-4 derived from tumor exosome can promote BNIP3L-dependent mitophagy and lactate production in CAFs, which in turn promotes the proliferation and EMT of cancer cells (46). Stromal nicotinamide N-methyltransferase (NNMT) expression is significant in functional aspects of the CAFs. It contributes to the depletion of S-adenosyl methionine and reduction in histone methylation, which regulate a series of gene expression associated with tumor progression (78). Glutamine-fructose-6-phosphate transaminase (GFPT)-2 is another prominent metabolism-regulating gene overexpressed in CAFs, which can drive the processes including glycolysis, pentose phosphate pathway, and TCA cycle process in cancer cells and promote tumor progression (79).

The mechanism of glucose metabolism is extensively studied in CAF–HCC cross-talk, while the processes like oxygen and lipid metabolism are rarely studied. The in-depth mechanisms underlying the metabolism regulation of CAF–HCC still warrant high-quality studies.

### CAFs Promote Angiogenesis in HCC

Tumor angiogenesis is an important dimension of HCC progression, characterized by the degradation of blood vessel basement membrane; the activation, proliferation, and migration of vascular endothelial cell; and the reconstruction of a new blood vessel. Proangiogenic stimulators including VEGF, FGF, PIGF, and TGF, as well as signaling pathways like TGF-β, EZH2/VASH1, and STEM/MEK/ERK, are extensively investigated (80–82). Multiple clinical studies have indicated the positive correlation between αSMA+ CAFs and neovascular formation (9, 80). Activated CAFs can release multiple angiogenic cytokines, including VEGF, MMP2, MMP9, bFGF, and TGF-β, and further contribute to HCC progression. Also, CD90 high expressed in CAFs may promote the release of PIGF, which can facilitate the angiogenesis and predict worse prognosis (80).

Regarded as a supplement to traditional angiogenesis theory, tumor vasculogenic mimicry (VM) serves as an important pattern for a rich tumor blood supply within the microenvironment. In this process, aggressive tumor cells generate their own blood supply channels without the participation of endothelial cells. Clinical studies have found that the rate of VM in HCC tissue is about 35% (83). The levels of CAF biomarkers α-SMA and vimentin and functional molecules MMP2 and EphA2 are highly correlated with VM in HCC patients (83). Furthermore, CAF-induced activation of TGF-β and SDF-1–CXCR4 signaling pathways plays significant roles in educating HCC cells expressing VE-cadherin, MMP2, and laminin, which can induce VM formation (84).

### CAFs Suppress Tumor Immunity in the HCC Microenvironment

As a critical frontline immune tissue, the liver is susceptible to the change of inflammation-related cells and cytokines in the microenvironment (85). The immunity components in HCC, including immunosuppressive regulatory T cells (Treg), tumor-associated macrophages (TAMs), tumor-associated neutrophils (TANs), pro-inflammatory CD4+/CD8+ T cells, natural killer (NK) cells, and dendritic cells (DCs), can be regulated by CAFs and their secreted factors (86). The tumor-expressed CD73 generates immunotolerant effect and promotes invasiveness *via* adenosine production from degradation of AMP in HCC (61, 87). In HCC, CAFs may activate c-Met and MEK–ERK1/2 signaling pathways, upregulate CD73 expression, and thereby promote the resistance to sorafenib and cisplatin (60). The CLCF-1–CXCL6/TGF-β axis plays an important role in the regulation of CAF-induced HCC stemness and immune evasion. HCC-derived CXCL6 and TGF-β stimulate ERK1/2 signal in CAFs, which upregulates the release of CLCF-1, forming a positive feedback loop between CAFs and HCC (21). It has been proven that activated HSCs can promote the apoptosis of effector T cells and NK cells and recruit Treg, causing harm to tumor immunity (88). Nicotinamide adenine dinucleotide phosphate oxidase (NOX)-4, a key regulator of CAF phenotype, can exclude CD8+ T-cell infiltration in HCC specifically (37). Inhibition of NOX4 can reverse CAFs to a quiescent state and restore CD8+ T-cell immunity, while blockade of TGF-β may prevent, rather than reverse, the activation of CAFs (37). NK cells can also be suppressed by the inhibitory factors PGE2 and IDO derived from CAFs (89).

In addition to the direct effects of CAFs on T cells and NK cells, they can also stimulate the IL-6/STAT3 pathway in neutrophils and further promote neutrophil proliferation, which impair T-cell function through the PD1/PD-L1 signaling pathway (90). Activated HSCs can enhance the activity of myeloid-derived suppressor cells (MDSCs) with increased iNOS and Arg-1 expression, which can inhibit the activation and function of effector T cells (88). Meanwhile, co-culture with activated HSCs can also stimulate the MDSCs through granulocyte–macrophage colony-stimulating factor (GM-CSF), M-CSF, COX-2, prostaglandin, and VEGF (91, 92).

M2-polarized macrophage is another prominent factor that impairs T-cell immunity through the deactivation of effector T cells and recruitment of Treg. High expression of CAF signature is associated with an immunosuppressive phenotype of infiltrating macrophage, suggesting the key role of CAF–macrophage interplay-mediated immunosuppression in the progression of HCC and fibrosis (27, 93). Studies have shown that TGF-β derived from macrophage can promote type I and III collagen expression in HSCs through the SMAD-dependent pathway (25, 94, 95). HSCs can also be activated by macrophage-derived cytokines including TGF-β, PDGF, oncostatin-M (OSM), tumor necrosis factor (TNF)-α, and IL-1β, as well as chemokines including CCL2 and CCL5 (94, 95). CAFs can in turn recruit monocytes *via* the SDF-1/CXCR4 pathway, which is associated with macrophage differentiation (96). CAFs can also inhibit pro-inflammatory features of M1-macrophages by reducing NO production and inhibiting pro-inflammatory cytokines (93). Meanwhile, CAFs promote M2 polarization of macrophage *via* release of SDF-1, IL-6, and GM-CSF and overexpression of PAI-1, producing an immunosuppressive tumor ecological niche (97, 98).

## The Metabolic Reprogramming of CAFs in HCC Progression

Tumor microenvironments are characterized as hypoxia and energy deficiency, which render cancer cells and other stromal cells to go through metabolic reprogramming (99), so as to meet their needs for increasing biological process of bioenergy, biosynthesis, and redox balance (100). The activation of CAFs in HCC also requires a large quantity of energy to promote cell proliferation, ECM and cytokine release, and the migration toward certain pro-fibrotic and pro-inflammatory regions (101). As a consequence, metabolic reprogramming and energy expenditure regulation play key roles in the function change of CAFs during the HCC microenvironment and liver injury. It has been proven that the metabolic reprogramming of CAFs in HCC includes changes in carbohydrate and mitochondrial metabolism, protein glycosylation, vitamin and lipid metabolism, and redox metabolism. HSCs are the main source of CAFs in HCC, and the metabolic pathways of HSC activation are in pivotal status (102).

The glycolysis process is active in cancer cells even in a rich oxygen surrounding, which is known as the Warburg effect (103). Due to the cross-talk between stromal cells and cancer cells, the Warburg effect has also been observed in fibroblasts, which is identified as the “reverse Warburg effect” (76). Studies have revealed that similar to cancer cells, the increased glucose utilization rate as well as aerobic glycolytic activity (104) and glucose transport capacity (105) has been discovered in activated primary HSCs and LX2 cell lines. Upregulation of glucose transporter proteins including GLUT-1, 2, and 4 (106) and increased expression of hexokinase 2 (107), fructose-2,6-bisphosphatase-3 (108), and pyruvate kinase (109) in HSCs also indicate that the HCC environment may educate HSCs toward abnormal glucose metabolism. Lactate shuttle is also an important phenomenon observed between cancer cells and CAFs (45). In this way, lactate produced from cancer cells can be utilized by CAFs in MCT4-dependent manners, which in turn generate pyruvic acid for tumor metabolism (76, 110). Within the lactate shuttle, more glucose prioritizing ATP production by CAF metabolism can meet the large energy requirement of cancer cells, which also represents the coordination between CAFs and cancer cells.

The increase of mitochondrial number and activity is also observed in activated CAFs in HCC, indicating an enhanced energy supplement from oxidative phosphorylation besides glycolysis (105). According to some studies, the consequence of stimulated oxidative phosphorylation is not limited to ATP production, while ROS generation and oxidative stress can be also caused by oxidative phosphorylation change (111, 112).

Besides carbohydrate, the reprogramming of lipid metabolism and amino acid metabolism in CAFs also provides major energy for ECM remodeling and tumor support. The storage of vitamin A and the regulation of lipid homeostasis are the main functions of quiescent HSCs in the liver (102). During HSC activation, the loss of retinyl ester-containing cytoplasmic droplets can be identified as the consequence of aberrant lipid metabolism (113, 114). Meanwhile, upregulated activity of fatty acid β oxidation in activated HSCs is required to metabolize lipid droplets which are derived from autolysosomes in HSCs (115, 116). During this process, the autophagy of HSCs and retinyl ester hydrolase activity are also enhanced (117).

The amino acid metabolic pattern in CAFs is characterized by glutaminolysis, which is the transition of glutamine toward a-ketoglutarate (118). An increased expression of glutaminase is observed in activated CAFs in HCC, leading to the metabolism of glutamine and the ensuing decreased peroxisome proliferator-activated receptor γ (PPARγ) expression and increased collagen release (119, 120). Significantly, a study has revealed that aberrant glutaminolysis in activated HSCs is accompanied by upregulated fibrogenesis (121).

## Some Types of CAFs May Exert a Tumor-Suppressive Effect

Although CAFs are mainly considered to support tumor growth, the antitumor functions of some subtypes of CAFs within a variety of cancers have been reported (13, 15, 17, 58, 122). The activated human fibroblast MRC5 is reported to induce apoptosis in the HCC LM3 cell line (58). Despite an EMT-enhancing effect, the apoptosis-inducing effect of fibroblasts provides new ideas and warrants further in-depth studies. Another recent study has shown the tumor-promoting effect of myofibroblastic CAF-secreted hyaluronan and inflammatory CAF-secreted HGF, which is responsible for the CAF–HCC interaction loop (17). On the other hand, not only cell contact and cell regulatory soluble factor but also the stiffness of the extracellular matrix remodeled by CAFs can also affect HCC development. Type I collagen expressed by myofibroblastic CAFs can inhibit HCC development by mechanically restraining tumor growth (17). This function may oppose the tumor-promoting effect of the CAF–HCC interaction loop. Another study indicated that exosome released from mesenchymal stem cell-derived CAFs contains miR-199a, which can sensitize HCC cells to the treatment of doxorubicin through the mTOR signaling pathway and reverse drug resistance in patients with advanced HCC (15).

Two CAF subtypes characterized by CD146 expression have distinct effects on tumor growth (122). The CD146− CAFs are reported to suppress estrogen receptor expression in breast cancer cells and increase the resistance to tamoxifen therapy, while CD146+ CAFs restore the sensitivity of cancer cells to tamoxifen treatment. The CD146− CAFs in tumor tissues predict worse patient outcomes, while CD146+ CAF is a positive prognostic index (122). Furthermore, in pancreatic ductal adenocarcinoma, the depletion of α-SMA+ myofibroblasts induced more aggressive and malignant tumor cells and enhanced the EMT process and cancer stemness (13). The tumor tissue-infiltrated CD4+ FOXP3+ Treg were also increased after CAF depletion, which reduced tumor response to gemcitabine treatment *via* cytotoxic T-lymphocyte-associated protein (CTLA)-4-induced immunosuppression (14). In another investigation related to CAFs in pancreatic ductal adenocarcinoma, sonic hedgehog is reported to form a fibroblast-rich desmoplastic stroma in a cancer environment. Once sonic hedgehog was depleted, unfavorable situations including undifferentiated histology, increased vascularity, and heightened proliferation were detected in the tumor (13). This suggested the antitumor function of sonic hedgehog and the ensuing fibroblast-rich stroma. This study also found that fibroblast can inhibit tumor angiogenesis through the hedgehog signaling pathway and prevent tumor progression.

The tumor-suppressive effect of some types of fibroblasts highlights the functional heterogenicity of CAF populations. When it comes to the development of CAF-targeting therapies, the positive role of this fibroblast subtype is supposed to be considered cautiously. Further studies are warranted to develop more reliable biomarkers in order to distinguish the distinct roles of CAFs.

## Traditional Chinese Medicines as Resources of Treating Liver Fibrosis

The pathogenesis of HCC is a complex process, in which a chronic inflammatory environment and fibrotic deposition play a key role. Multiple strategies have been developed to attenuate liver fibrosis and exert antitumor effects (6, 7, 36). Traditional Chinese medicine has been proven effective in delaying tumor progression and preventing the recurrence and metastasis of HCC (123, 124). Recently, the antifibrotic effects of Chinese medicine decoction and natural product extracts have been extensively studied. Many studies have suggested that the antifibrotic and HCC-preventive effect of TCM components lies in the medicines with “qi-soothing in liver”, “blood stasis-removing”, and “heat-clearing and detoxifying” efficacy. Multiple medicines including *Radix Salviae miltiorrhizae*, *Astragalus mongholicus*, *Radix bupleuri*, *Paeoniae radix rubra*, *Atractylodes macrocephala*, *Paeonia Lactiflora*, *Trionycis Carapax*, and *Angelica sinensis* are commonly used clinically to achieve therapeutic goals (125). Several clinical studies have proven the effectiveness of TCM in preventing liver fibrosis and HCC recurrence after radical surgery. Huaier granule, an aqueous extract of *Trametes robinophila* Murr-based medicine approved by the Chinese State Food and Drug Administration, has been found to efficiently prolong recurrence-free survival and reduce extrahepatic recurrence in patients with HCC who underwent curative resection (126, 127). Jiedu granule, which is composed of four traditional Chinese herbal medicines including *Actinidia valvata*, *Salvia chinensis*, *Cremastra appendiculata*, and the gizzard membrane of *Gallus gallus domesticus*, has been proven effective in preventing recurrence as an adjuvant therapy with cinobufacini injection for HCC after curative resection (128, 129). Another randomized controlled trial revealed that Chunggan extract, an herbal formula, can ameliorated hepatic fibrosis and liver stiffness in patients with chronic liver disease (130). Liver fibrosis is a significant factor mediating recurrence after radical surgery, which can be attenuated by TCM administration in both single drug application and drug combination. The values of TCM in liver fibrosis treatment are supposed to lie in the prevention of cirrhosis–HCC transition and HCC progression; alleviation of symptoms in advanced HCC, which can be mediated by the regulation of CAFs/HSC activation; the suppression of hepatic inflammation; the improvement of antioxidant capacity; and the inhibition of vasculogenic mimicry (131).

The most prominent signaling pathway involved in the antifibrotic effect of TCM is TGF-β (25, 132). Total flavanones extracted from *Sedum sarmentosum* can suppress the activation of HSCs *via* the TGF-β1/SMAD pathway (133). Methyl ferulic acid, which is the primary monomer constituent of herbal medicines including *Ferula assafoetida* L., *Ligusticum chuanxiong* Hort., and *Lycopodium selago* L., can alleviate the progression of liver fibrosis through HSC-suppressing function *via* the TGF-β1/SMAD and NOX4/ROS signaling pathways (47). Salvianolic acid A, derived from *Alvia miltiorrhiza* Bge., can inhibit TGF-β and PI3K/AKT/mTOR signaling cascades in CCl-4-induced fibrosis mice, improve fibrotic degree, restore liver function, and reduce collagen deposition in the liver (134). The Xiaoyaosan and Yinchenhao decoction can inhibit the activation of HSCs *via* the TGF-β and Akt/FoxO3 pathways (135, 136). Yinchenhao decoction can also promote the apoptosis of parenchyma cells in a TNF-involved manner, which blocks the initiation of HCC in a cirrhosis environment (136). Besides medical treatment, *Ammophila Arenaria umbilical* moxibustion, a form of manual therapy, can promote the apoptosis of activated HSCs and prevent the progression of HCC (137). NF-κF and PPARγ are the other two key factors regulating TGF-β signaling and, thus, the activation of HSCs (138). In liver fibrosis, M2-polarized macrophage can bolster the transformation of HSCs to CAFs by extensively activating NF-κb signaling (139). Otherwise, the suppression of NF-κB and stimulation of PPARγ can inhibit the expression of TGF-β1. *Ginkgo biloba* extract (140, 141), 18α-glycyrrhizin (142), glycyrrhizic acid (143), and curcumin (144) can also suppress NF-κB signaling in the liver and alleviate liver fibrosis. Baicalin (145) and curcumin (146) can exert a protective effect on CCl4-induced liver injury by activating PPARγ and inhibiting TGF-β.

Ferroptosis is a novel pathway of cell death which is widely investigated in recent years. A study showed that *Magnesium isoglycyrrhizinate* attenuates fibrotic scar formation in CCl4-induced hepatic–fibrotic rats through ferroptosis-related cell death of HSCs (147). Artemether, extracted from *Artemisia apiacea*, promotes the accumulation of iron and lipid peroxides and, thus, induces ferroptosis in HSCs (148). This study also showed that tumor suppressor p53 plays a key role in artemether-induced HSC ferroptosis (148). In addition, iron regulatory protein 2 accumulation is another key mechanism of artemether-mediated antihepatic fibrosis process (149).

Activated CAFs can facilitate the development of HCC *via* stemness and autophagy induction, while chloroquine can downregulate stemness markers including Nanog, Sox2, and Oct4 in CAFs co-cultured with HCC cells, subsequently modulating the autophagy of cancer cells (150). GPR68 is a key protein regulating the proliferation and invasion of HCC cells. Conophylline can suppress the expression of GPR68 in CAFs and block the tumor-promoting effect of CAFs (52). A clinical study confirmed the therapeutic advantage of combined conophylline and sorafenib treatment in advance HCC (52). Exosomal circCCT3 derived from CAFs can stimulate the activity of glucose metabolism-related gene HK2 and further affect the glucose metabolism of HCC cells (151), which is blocked by coptisine, the main component of *Chelidonium majus* L., *Corydalis uanhusuo* W. T. Wang, and *Coptis japonica* Makino (152). Glycyrrhizin and its metabolites glycyrrhetinic acid can attenuate non-alcoholic steatohepatitis-induced liver fibrosis, which is mediated by suppression of NLR family pyrin domain-containing 3 (NLRP3) inflammasome and restoration of bile acid homeostasis (153). The CAF-induced immunosuppressive microenvironment is involved in HCC progression to a large extent, which can be improved by phillygenin derived from *Forsythiae fructus* (154). The expression of immunosuppressive IL-6, IL-1β, and TNF-α by CAFs can be inhibited by phillygenin treatment through the TLR4/MyD88/NF-κB signaling pathway (154).

The advantages of TCM treatment in liver fibrosis lie in the comprehensive treatment of multisite and multitarget conditions and overall regulation. Several extracts of Chinese medicine and their derivatives equipped with antifibrotic property are summarized in **Table 2**. The development of cirrhosis and the initiation of HCC are complex processes referring to numerous signaling pathway activation and multiple functional changes, making it possible for TCM to exert a multitarget–regulation effect.

**Table 2:** Chinese medicine and their derivatives equipped with antifibrotic property.

Drugs	Categories	Mechanisms	Signaling pathways	Phenotypes	Cells or animal models	Reference
Chloroquine	Monomer	LC3-II protein accumulation		Stemness, invasion, and metastasis ↓ of CAF-CM-treated Huh7	CAFs	(150)
Curcumin	Herbal extract	CTGF suppression	NF-κB and ERK	Type I collagen ↓ in HSC	Primary HSCs	(155)
		PPARγ stimulation	PPARγ-ROS; PDGF and EGF signaling	Oxidative stress, inflammation ↓; α-SMA and collagen I ↓ in HSC; serum AST, ALT, and ALP ↓; liver histological architecture improved	CCl4-induced liver fibrotic rats; primary HSCs	(156–159)
α-Lipoic acid; N-acetylcysteine	Monomer	Oxidative stress reduction and MMP13 induction	TGF-α signaling	Collagen deposition ↓; serum AST, ALT, and γ-GT ↓; GSH ↑	CCl4-induced liver fibrotic rats	(156)
Magnesium isoglycyrrhizinate	Herbal extract	Heme oxygenase-1 stimulation	Heme oxygenase-1/ferrostatin-1	Fibrotic scar formation ↓; ferroptosis-related cell death of HSC	CCl4-induced liver fibrotic rats	(147)
Artemether	Monomer	Tumor-suppressor p53 stimulation		Liver injury and fibrotic scar formation ↓; ferroptosis-related cell death of HSC	CCl4-induced liver fibrotic mice; primary HSCs	(148)
		IRP2 accumulation	IRP2–iron–ROS axis	Production of iron and ROS ↑ in HSC; ferroptosis-related cell death of HSC	Activated LX2	(149)
Osthole	Monomer	Nuclear translocation of p65 suppression; oxidative stress reduction and inflammation inhibition	TGF-β1/NF-κB	Plasma AST and ALT ↓; histological architecture and hepatic fibrosis scores improved; collagen and α-SMA ↓	Thioacetamide-induced liver fibrotic rats; activated HSC-T6 and LX2	(160)
*Ginkgo biloba* extract	Herbal extract	Oxidative stress reduction; NF-κB p65 suppression	TGF-β1/NF-κB	Serum ALT, AST, HA, and LN ↓; histopathologic scores of liver fibrosis ↓; collagen I and α-SMA ↓	CCl4-induced liver fibrotic rats; primary HSCs	(161, 162)
18α-Glycyrrhizin; glycyrrhetinic acid; glycyrrhizin	Monomer	Blockade of the translocation of NF-κB	NF-κB	α-SMA ↓ in HSC; HSC apoptosis; procollagen I and III ↓	CCl4-induced liver fibrotic rats; primary HSCs	(163, 164)
		NLRP3 inhibition; inflammation-mediated hepatic farnesoid X receptor inhibition; restoration of bile acid homeostasis		Hepatic steatosis, inflammation, and fibrosis improved; serum bile acid accumulation ↓	Methionine- and choline-deficient diet-induced NASH mice model	(153)
Baicalin	Monomer	TGF-β1 suppression; PPARγ stimulation		HSC activation ↓	CCl4-induced liver fibrotic rats	(145)
Silymarin	Monomer	CTGF and hepatic hydroxyproline decrease; SOD and GPx increase		Plasma AST and ALT ↓; hepatic fibrosis formation ↓	CCl4-induced liver fibrotic rats	(165)
*Boswellia serrata* and *Salvia miltiorrhiza* combination	Herbal extract	CTGF, TGF-β1, Smad3, and Smad7 reduction	TGF-β signaling	Histological severity of fibrosis improved; α-SMA ↓; collagen I, II, and III ↓	DMN-induced liver fibrotic rats	(166)
*Sedum sarmentosum* total flavanones	Herbal extract	Smad2/3, Smad4 decrease; Smad7 increase	Smads pathway	Serum ALT, AST, HA, and LN ↓; α-SMA ↓	CCl4-induced liver fibrotic rats	(133)
Methyl ferulic acid	Monomer		TGF-β1/Smad and NOX4/ROS	HSC proliferation ↓; α-SMA and collagen I ↓	Activated LX2	(47)
Praziquantel	Monomer	Smad7 increase	TGF-β/Smad signaling	Activation of HSC ↓; collagen matrix ↓	CCl4-induced liver fibrotic mice; activated HSC-LX2, fibroblast-like cell line MES13, and fibroblast cell line NIH3T3	(167)
Conophylline	Alkaloid	G protein-coupled receptor 68 suppression		α-SMA ↓ in CAFs; cancer-promoting effects of CAFs ↓; cytokines including IL-6, IL-8, CXCL2, angiogenin, and OPN ↓ produced by CAFs	Primary CAFs	(52)
Coptisine	Alkaloid	Blocked secretion of CAF-derived exosomal circCCT3; changed glucose metabolism in HCC cells	circCCT3/HK2	Cell viability and metastasis ↓ of CAF-CM-treated Huh7 and HepG2	Primary CAFs; clinical HCC tumors	(151, 152)
Salvianolic acid A	Monomer	Apoptosis of HSC	PI3K/AKT/mTOR; Bcl-2/Bax; caspase-3/cleaved caspase-3	Liver fibrosis index ↓; collagen deposition ↓; activation of HSC ↓; synthesis of extracellular matrix ↓	CCl4-induced liver fibrotic rats; primary CAFs	(134)
Xiaoyaosan	Decoction	p-FoxO3a, p-Smad3, and p-Akt decrease	TGF-β/Smad and Akt/FoxO3	Liver function improved; fibrotic changes alleviated; α-SMA and collagen I ↓	CCl4-induced liver fibrotic rats; activated LX2	(135)
Yinchenhao decoction	Decoction	Cleaved caspase-3 decrease in L02 cells; p-ERK1/2, PI3K, and Bcl-XL protein increase in L02 cells; Bax and cleaved caspase-8 increase in LX2 cells	Apoptosis-related signaling	Liver fibrosis improved; apoptosis of hepatic parenchyma cells ↓; LX2 cell proliferation ↓	DMN-induced liver fibrotic rats; human hepatic L02 cells and activated LX2 cells	(136)
Phillygenin	Monomer	TLR4, MyD88, IKKβ, p65, IκBα, and TAK1 molecular docking	TLR4/MyD88/NF-κB	Collagen I and α-SMA ↓ in LX2; IL-6, IL-1β, and TNF-α ↓ in LX2	Activated LX2 cells	(154)
*Forsythiae fructus*	Herbal extract		TLR4/MyD88/NF-κB and TGF-β/Smads signaling	Hepatic histopathological injury, abnormal liver function, fibrosis, and inflammation improved; α-SMA and collagen-1 ↓	CCl4-induced liver fibrotic rats	(168)

CAF, cancer-associated fibroblasts; CM, conditioned medium; CTGF, connective tissue growth factor; NF-κB, nuclear factor-κB; HSC, hepatic stellate cell; PPARγ, peroxisome proliferator-activated receptor γ ; ROS, reactive oxygen species; PDGF, platelet-derived growth factor; EGF, epidermal growth factor; α-SMA, α-smooth muscle actin; AST, aspartate aminotransferase; ALT, alanine aminotransferase; ALP, alkaline phosphatase; CCL4, carbon tetrachloride; MMP, matrix metalloproteinase; TGF, transforming growth factor; γ-GT, γ-glutamyl transferase; GSH, L-glutathione; IRP2, iron regulatory protein 2; HA, hyaluronic acid; LN, laminin; NLRP3, nucleotide-binding and oligomerization domain-like receptor containing pyrin domain containing 3; NASH, non-alcoholic steatohepatitis; SOD, superoxide dismutase; DMN, N-nitrosodimethylamine; Bcl-2, B-cell lymphoma-2; Bax, BCL2-associated X; IL, interleukin; TNF, tumor necrosis factor; TLR, Toll-like receptors. ↑：upregulation, ↓：downregulation.

## Conclusion

The role of CAFs in HCC initiation and progression should not be ignored. The unique premalignant microenvironment in fibrosis tends to lead to the activation and tumor-supporting function of CAFs, which can be reversed or blocked by the application of TCM. HSCs are one of the dominant sources of CAFs in HCC. Generally, inactivated HSCs are quiescent in normal liver and equipped with the following functions significant for the maintenance of normal liver function: the metabolism and storage of vitamin A, the storage of lipid, and the production and secretion of ECM components including collagen and glycoprotein. Once stimulated by inflammatory factors and mechanical injuries in pathogenic conditions, the HSCs can be transformed to activated CAFs, which display myofibroblast-like phenotypes and highly express α-SMA, vimentin, desmin, etc. Meanwhile, the proliferation and mobility properties of HSCs can be enhanced to promote the migration toward the damage sites. Under precancerous conditions, the ECM can also be remodeled with increased release of ECM components and decreased decomposition capacity. Furthermore, the remodeled ECM can in turn facilitate the activation of CAFs and HCC progression by unfavorable matrix stiffness and contractile activity. The upregulated cytokines, chemokines, and receptor/ligand binding can also affect fibrosis and tumor progression.

The mechanisms for CAFs in supporting HCC progression can be categorized into upregulated aggressiveness and stemness, transformed metabolism toward glycolysis and glutamine reductive carboxylation, polarized tumor immunity toward immune escape of HCC cells, and increased angiogenesis. The TGF-β/SMAD signaling pathway plays a dominant role in all stages of liver fibrosis including the activation of HSCs, the deposition of ECM, the aggressiveness and stemness generation of CAF-educated HCC cells, and the process of CAF-induced angiogenesis. In the CAF activation process, TGF-β1 inhibits the apoptosis of normal fibroblasts and induces the excessive synthesis of collagen and fibronectin. The phosphorylation of SMAD2/3 is a canonical mechanism involved in the activation of CAFs. Besides the TGF-β1/SMAD pathways, TGF-β1 can also stimulate downstream MAPK, NF-κB, and PI3K to activate CAFs and generate fibrosis.

TCM plays a key role in fibrosis and cancer treatment. Both clinical and fundamental studies have verified the unique properties of TCM in its antifibrosis application. Based on the findings presented in this review, TCM can be regarded as a potential and potent strategy for the prevention and treatment of both liver fibrosis and liver cancer.

## Author Contributions

WJ and SL drafted the manuscript with the support from CL and BC. BC revised the manuscript. All authors contributed to the article and approved the submitted version.

## Funding

This study was supported by the grants from the National Natural Science Foundation of China (Nos. 82074203 and 82030117).

## Conflict of Interest

The authors declare that the research was conducted in the absence of any commercial or financial relationships that could be construed as a potential conflict of interest.

## Publisher’s Note

All claims expressed in this article are solely those of the authors and do not necessarily represent those of their affiliated organizations, or those of the publisher, the editors and the reviewers. Any product that may be evaluated in this article, or claim that may be made by its manufacturer, is not guaranteed or endorsed by the publisher.
